# Supplementary material for: Ecology of Endozoicomonadaceae in three coral genera across the Pacific Ocean
Source: Nat Commun. 2023 Jun 1;14:3037. doi: 10.1038/s41467-023-38502-9 (PMC10235432; doi:10.1038/s41467-023-38502-9)
Supplement: Supplementary file 1 — Supplementary Information [file 41467_2023_38502_MOESM1_ESM.pdf]

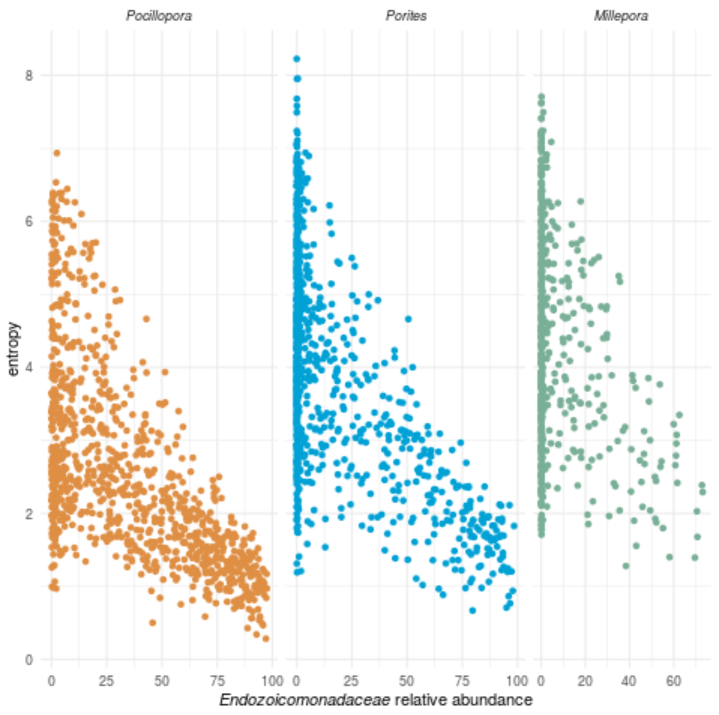

a.

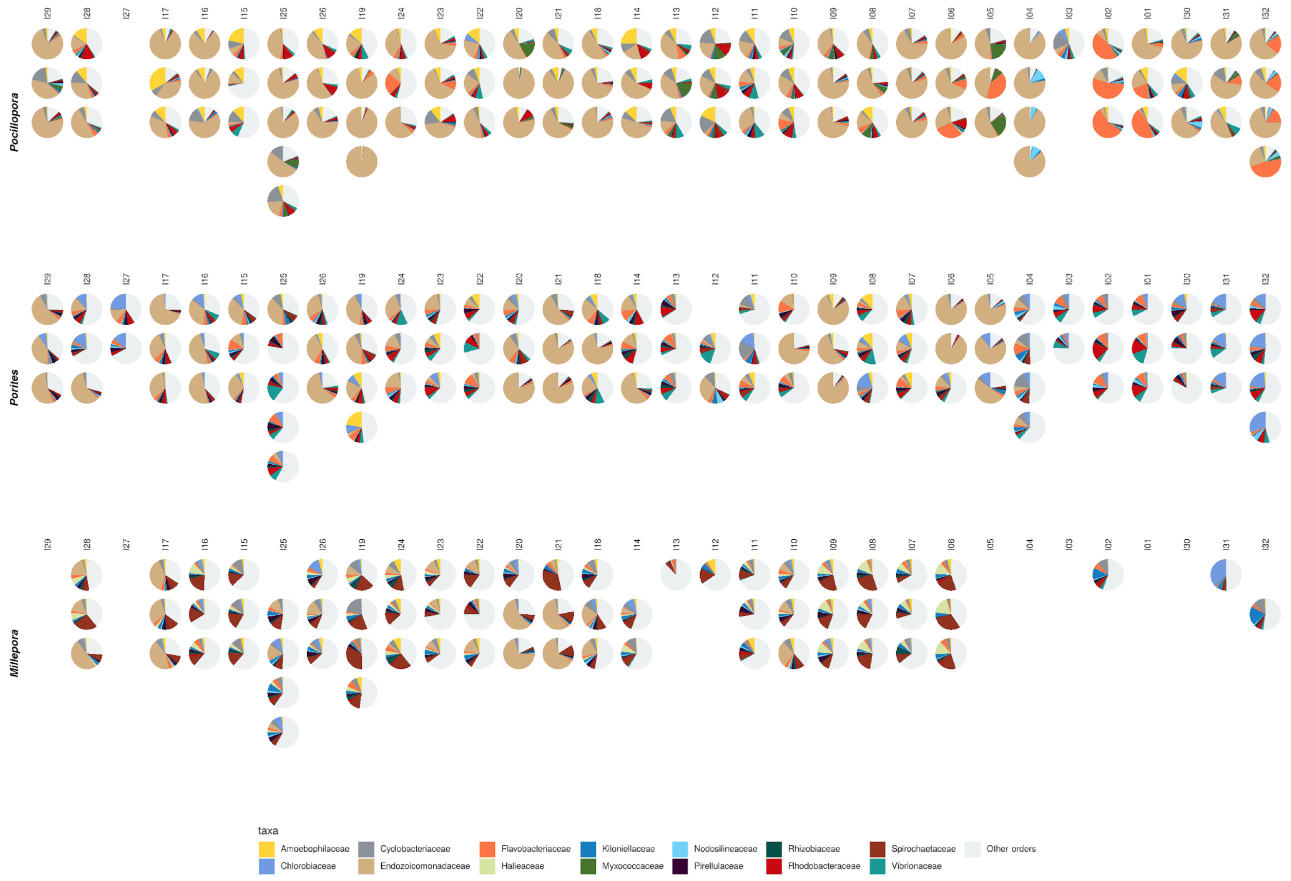

b.

Supplementary Fig. 1. a. Shannon diversity (entropy) of *Endozoicomonadaceae* communities in *Pocillopora*, *Porites* and *Millepora* in relation to *Endozoicomonadaceae* relative abundance. **b**. Relative abundance of the most abundant bacteria families averaged per site.

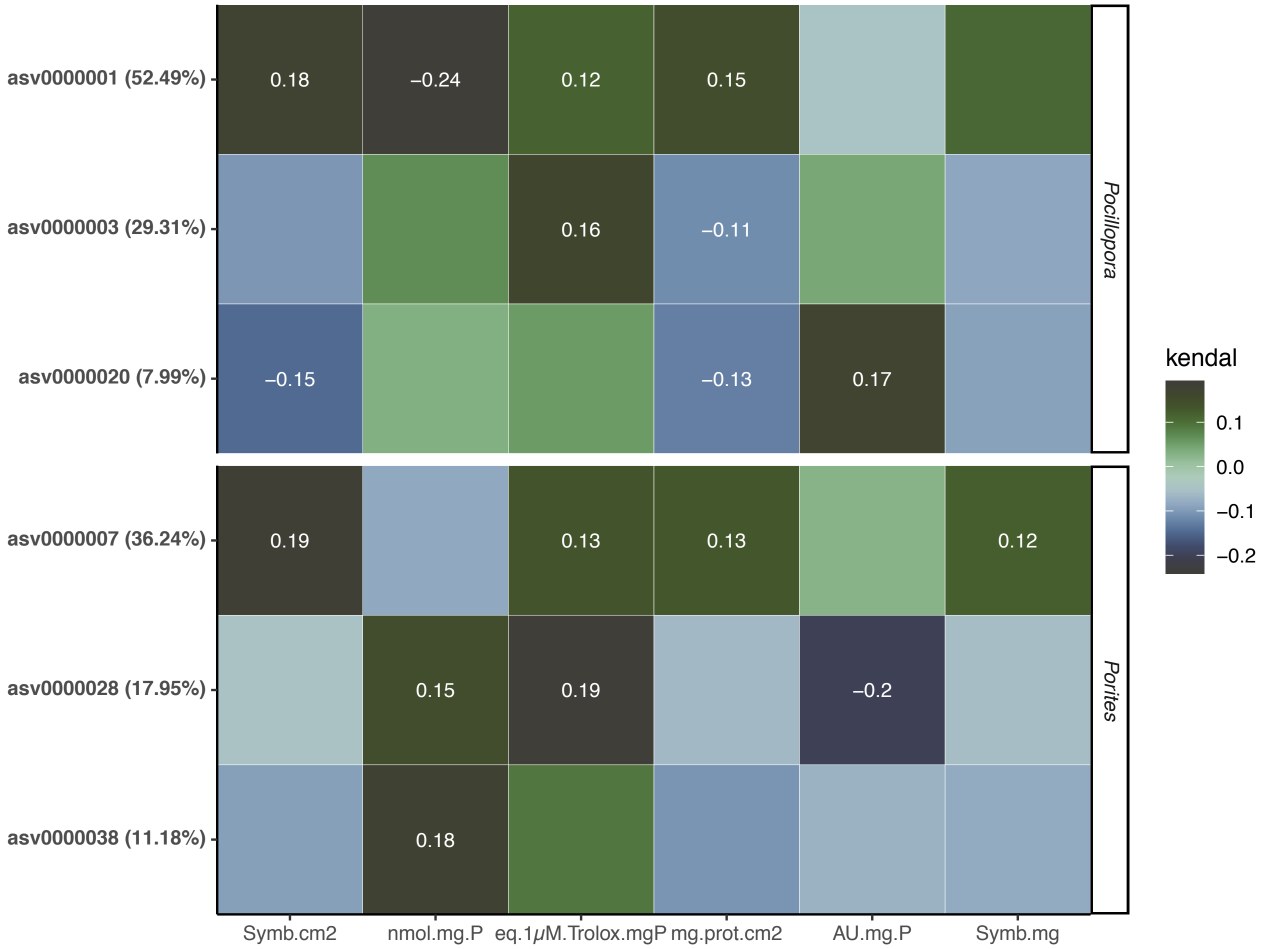

Supplementary Fig. 2. Kendall correlations between coral phylotypic biomarkers and ASV abundance in *Pocillopora* and *Porites*. Symb.cm2: *Symbiodiniaceae* content per coral surface, nmol.mg.P: protein carbonylation per coral mg of protein, eq.1uM.Trolox.mgP: total antioxidant content per coral mg of protein, mg.prot.cm2: protein content per coral surface, Au.mg.P: protein ubiquitination per coral mg of protein, Symb.mg: *Symbiodiniaceae* content per coral mg of protein.

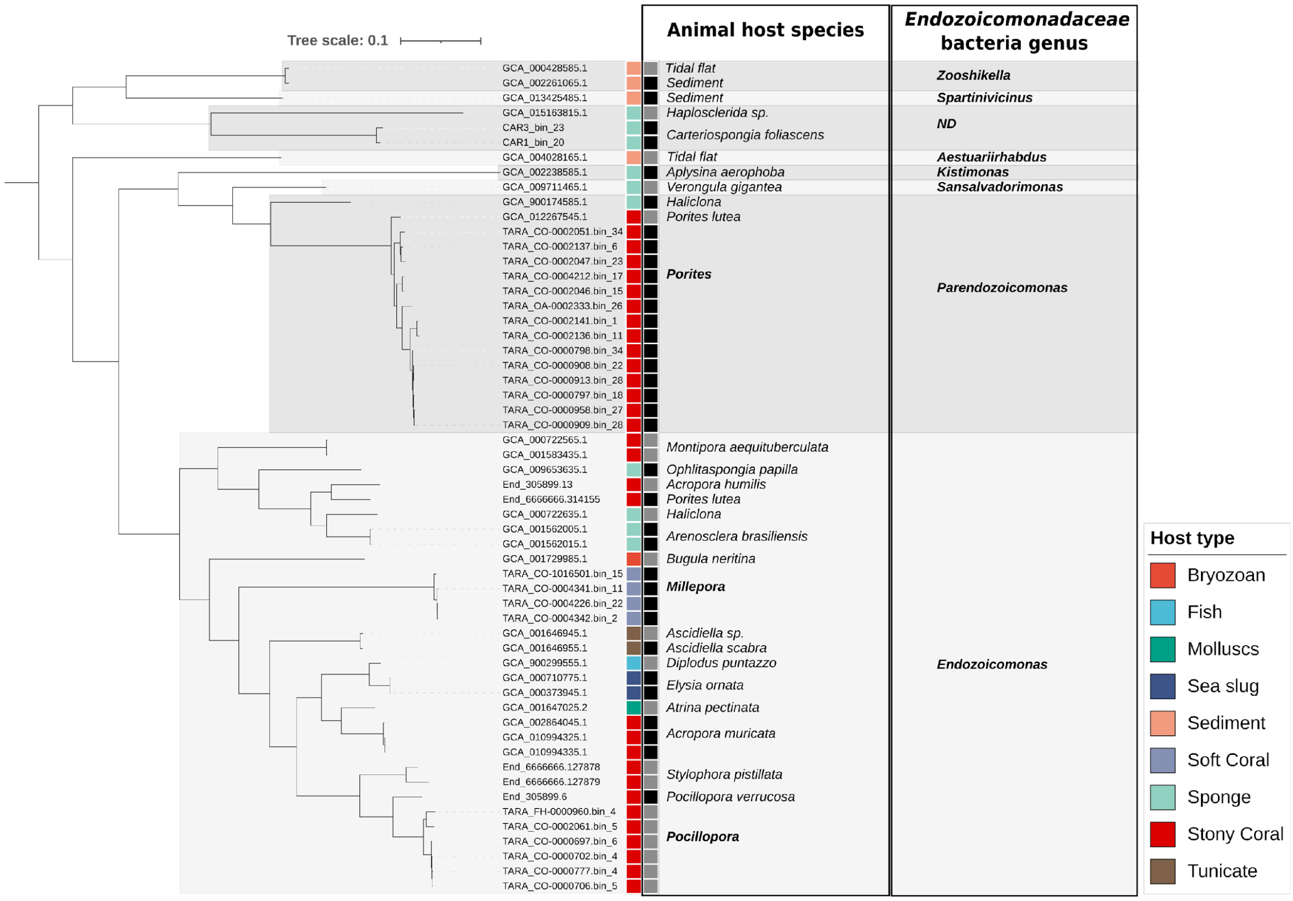

Supplementary Fig. 3. Distance tree of 71 concatenated genes from MAGs obtained from the Tara Pacific expedition and references from the literature. MAGs from this study have TARA_ in their name. The origin of the MAGS is indicated by a colour code. The black and grey code differentiate the different hosts.

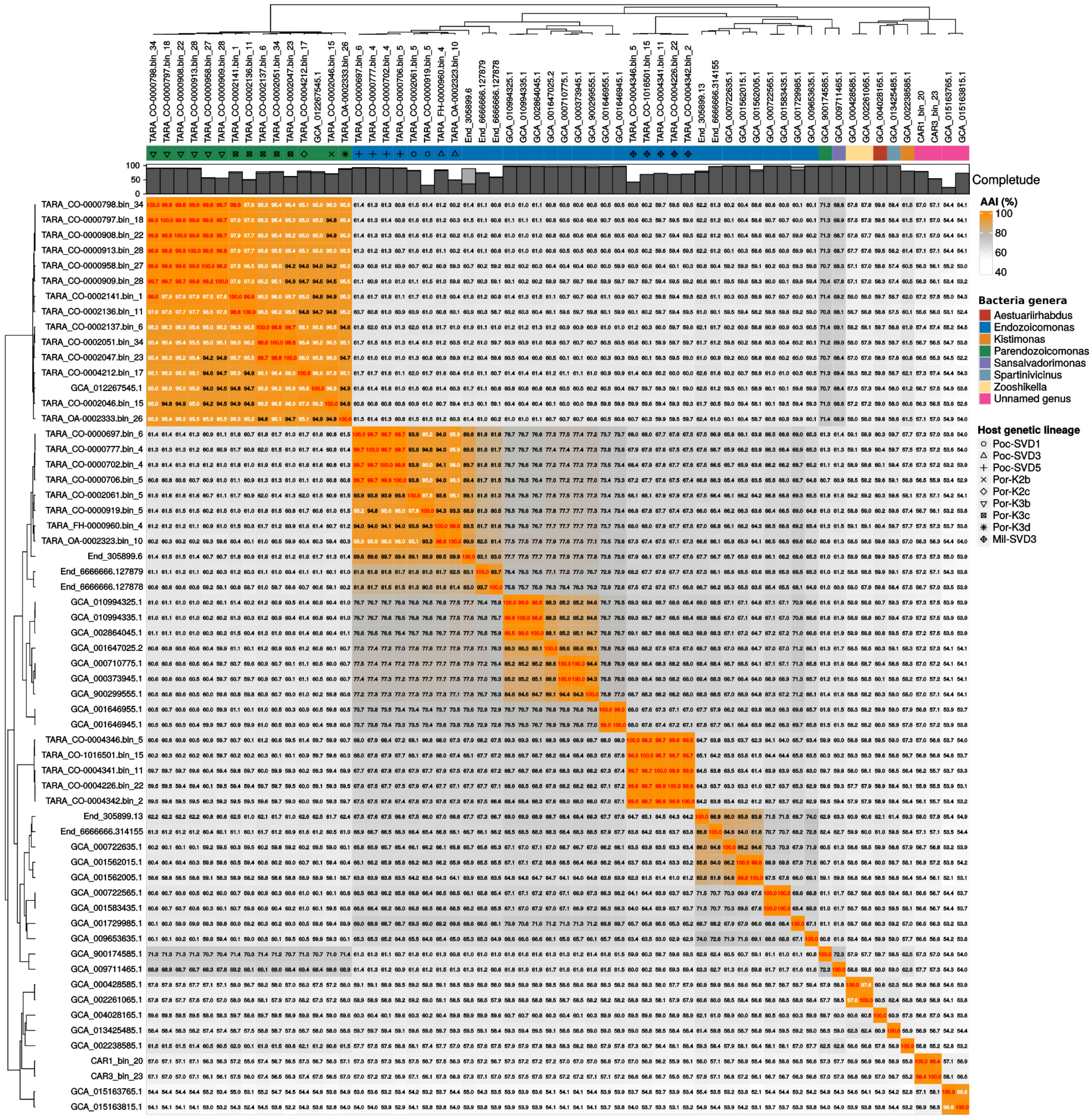
Completude
AAI (%)
100
80
60
40
Bacteria genera
Aestuariirhabdus
Endozoicomonas
Kistimonas
Parendozoicomonas
Sansalvadorimonas
Spartinivicinus
Zooshikella
Unnamed genus
Host genetic lineage
Poc-SVD1
Poc-SVD3
Poc-SVD5
Por-K2b
Por-K2c
Por-K3b
Por-K3c
Por-K3d
Mil-SVD3
TARA_CO-0000798.bin_34
TARA_CO-0000797.bin_18
TARA_CO-0000908.bin_22
TARA_CO-0000913.bin_28
TARA_CO-0000958.bin_27
TARA_CO-0000909.bin_28
TARA_CO-0002141.bin_1
TARA_CO-0002136.bin_11
TARA_CO-0002137.bin_6
TARA_CO-0002051.bin_34
TARA_CO-0002047.bin_23
TARA_CO-0004212.bin_17
GCA_012267545.1
TARA_CO-0002046.bin_15
TARA_OA-0002333.bin_26
TARA_CO-0000697.bin_6
TARA_CO-0000777.bin_4
TARA_CO-0000702.bin_4
TARA_CO-0000706.bin_5
TARA_CO-0002061.bin_5
TARA_CO-0000919.bin_5
TARA_FH-0000960.bin_4
TARA_OA-0002323.bin_10
End_305899.6
End_6666666.127879
End_6666666.127878
GCA_010994325.1
GCA_010994335.1
GCA_002864045.1
GCA_001647025.2
GCA_000710775.1
GCA_000373945.1
GCA_900299555.1
GCA_001646955.1
GCA_001646945.1
TARA_CO-0004346.bin_5
TARA_CO-1016501.bin_15
TARA_CO-0004341.bin_11
TARA_CO-0004226.bin_22
TARA_CO-0004342.bin_2
End_305899.13
End_6666666.314155
GCA_000722635.1
GCA_001562015.1
GCA_001562005.1
GCA_000722565.1
GCA_001583435.1
GCA_001729985.1
GCA_009653635.1
GCA_900174585.1
GCA_009711465.1
GCA_000428585.1
GCA_002261065.1
GCA_004028165.1
GCA_013425485.1
GCA_002238585.1
CAR1_bin_20
CAR3_bin_23
GCA_015163765.1
GCA_015163815.1

a.

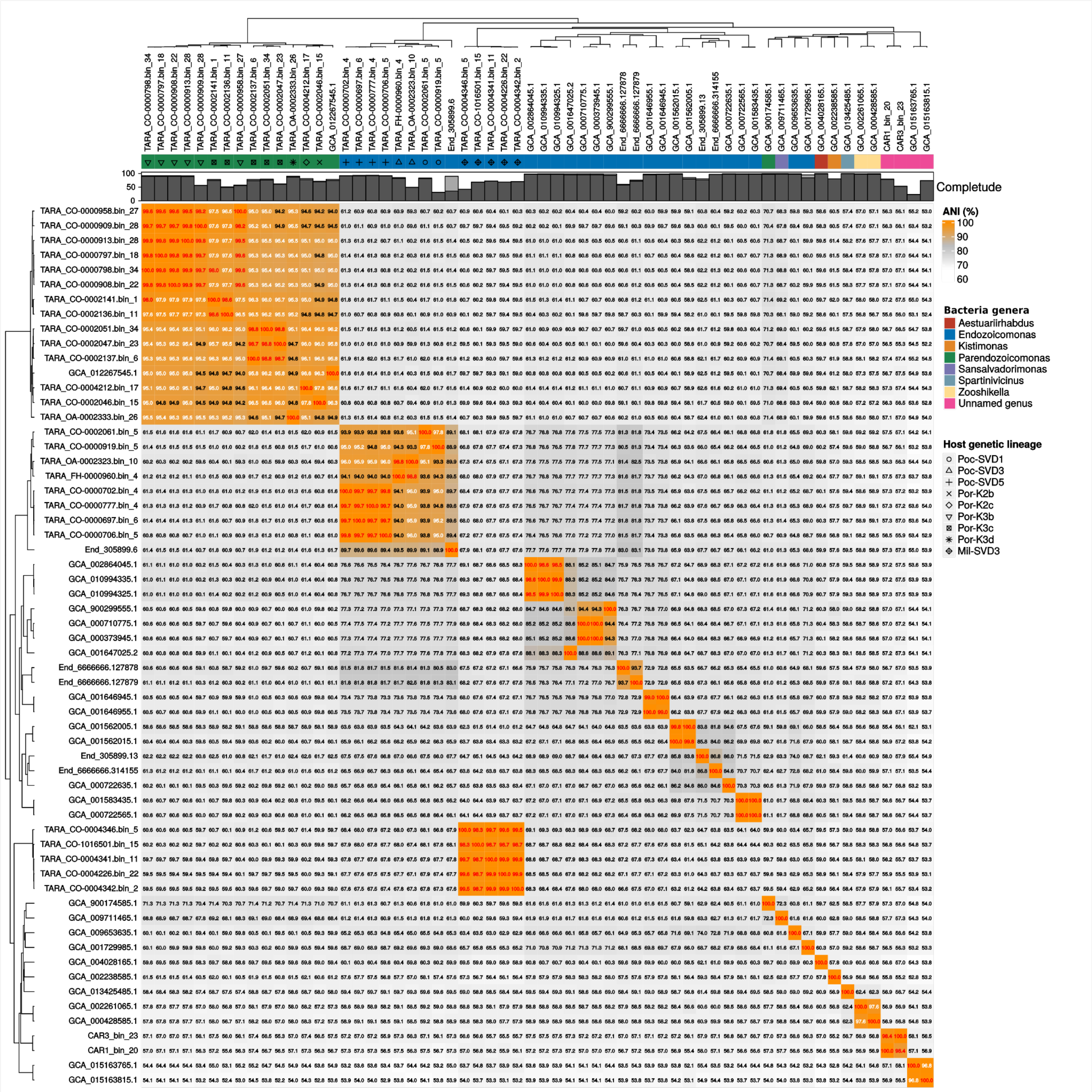

b.

Supplementary Fig. 4. Heatmap showing the pairwise average amino acid identities (AAI) (a) and average nucleotid identity (AAI)(b) between MAGs obtained in this study (TARA_) and others from the literature. Completude, GC content, origin of the MAGs are indicated. Geometric symbols differentiate the corals' genetic lineages. MAGs are grouped according to their phylogeny.

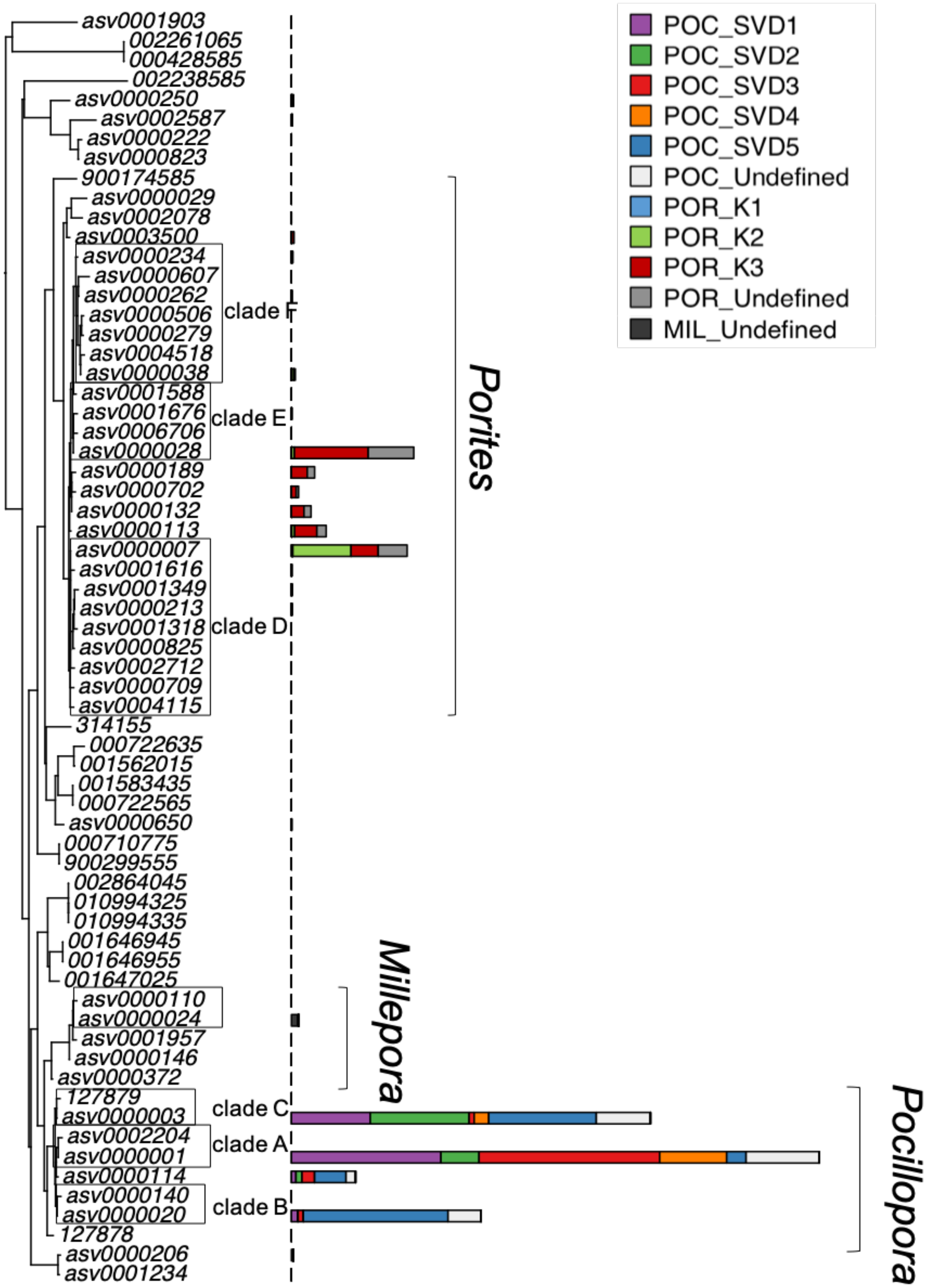

Supplementary Fig. 5. Distance tree of the most abundant *Endozoicomonadaceae* 16S rRNA ASVs found in *Pocillopora*, *Porites* and *Millepora*. The proportion of the coral host genetic lineage in which they were found is indicated as barre charts.

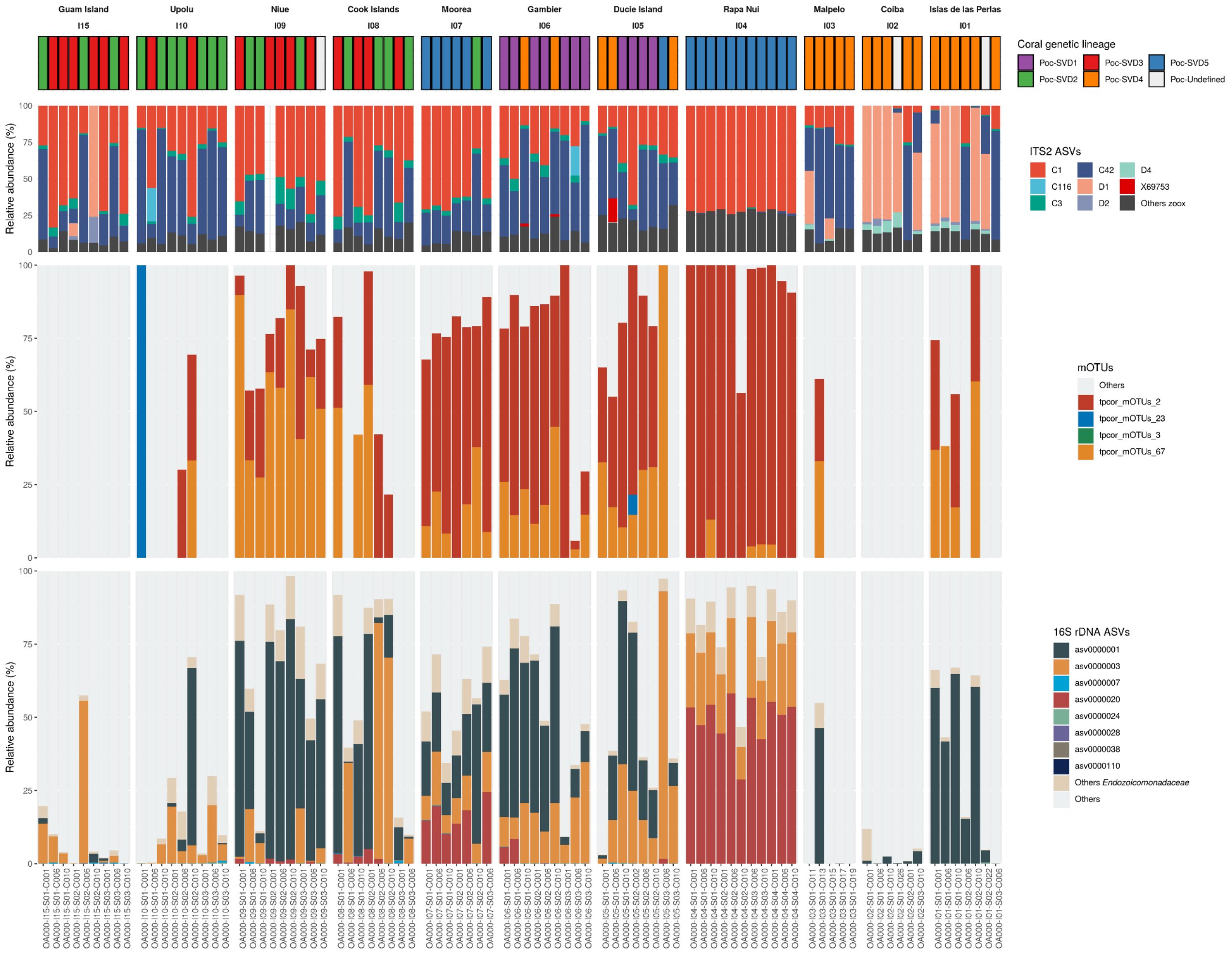
Guam Island
I15
Upolu
I10
Niue
I09
Cook Islands
I08
Moorea
I07
Gambier
I06
Ducie Island
I05
Rapa Nui
I04
Malpelo
I03
Coiba
I02
Islas de las Perlas
I01
Coral genetic lineage
Poc-SVD1
Poc-SVD2
Poc-SVD3
Poc-SVD4
Poc-SVD5
Poc-Undefined
ITS2 ASVs
C1
C116
C3
C42
D1
D2
D4
X69753
Others zoox
mOTUs
Others
tpcor_mOTUs_2
tpcor_mOTUs_23
tpcor_mOTUs_3
tpcor_mOTUs_67
16S rDNA ASVs
asv0000001
asv0000003
asv0000007
asv0000020
asv0000024
asv0000028
asv0000038
asv0000110
Others Endozoicomonadaceae
Others
Relative abundance (%)
0
25
50
75
100

a.

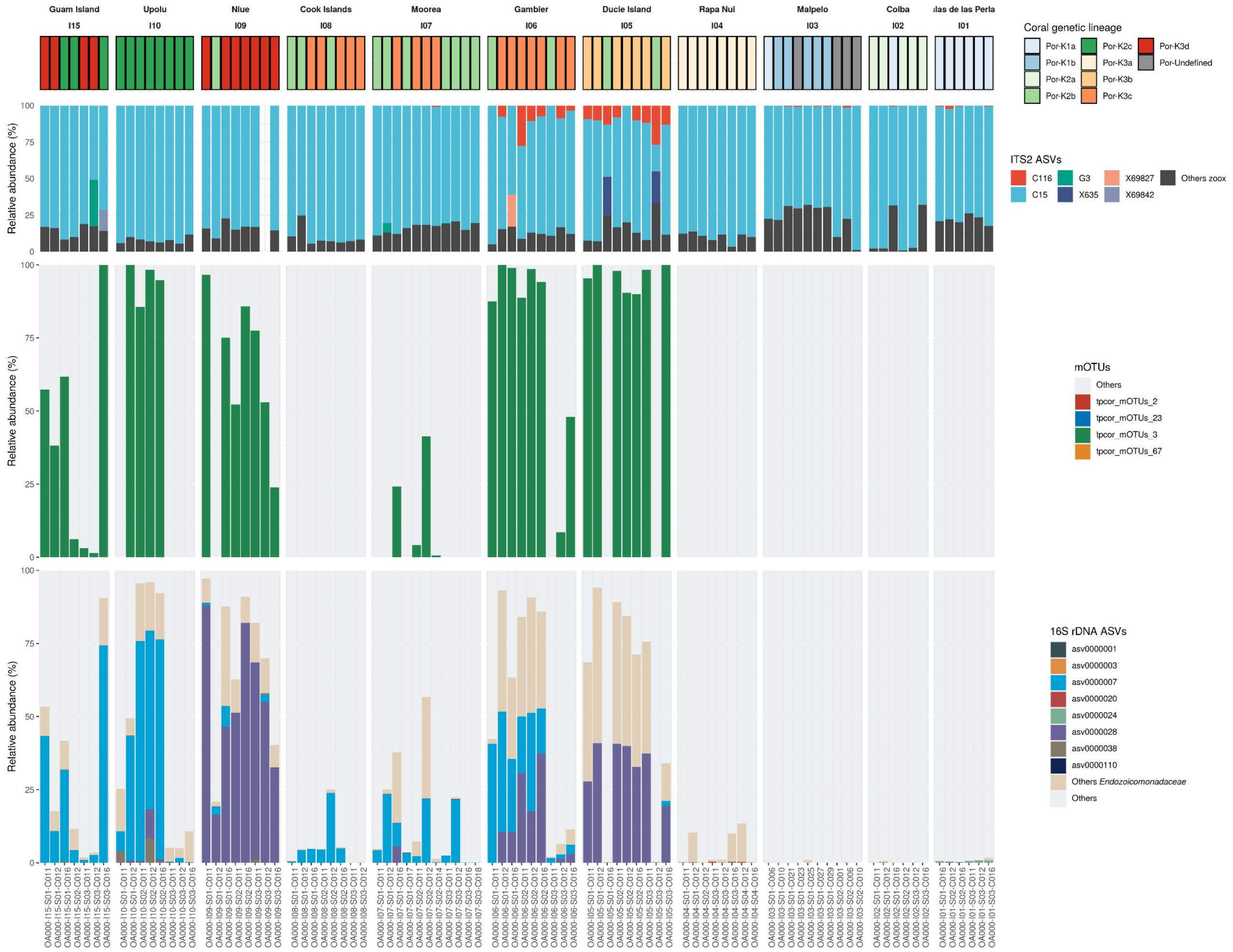
Guam Island
I15
Upolu
I10
Niue
I09
Cook Islands
I08
Moorea
I07
Gambier
I06
Ducie Island
I05
Rapa Nui
I04
Malpelo
I03
Coiba
I02
Islas de las Perlas
I01
Coral genetic lineage
Por-K1a
Por-K1b
Por-K2a
Por-K2b
Por-K2c
Por-K3a
Por-K3b
Por-K3c
Por-K3d
Por-Undefined
ITS2 ASVs
C116
C15
G3
X635
X69827
X69842
Others zoox
mOTUs
Others
tpcor_mOTUs_2
tpcor_mOTUs_23
tpcor_mOTUs_3
tpcor_mOTUs_67
16S rDNA ASVs
asv0000001
asv0000003
asv0000007
asv0000020
asv0000024
asv0000028
asv0000038
asv0000110
Others Endozoicomonadaceae
Others
Relative abundance (%)

b.

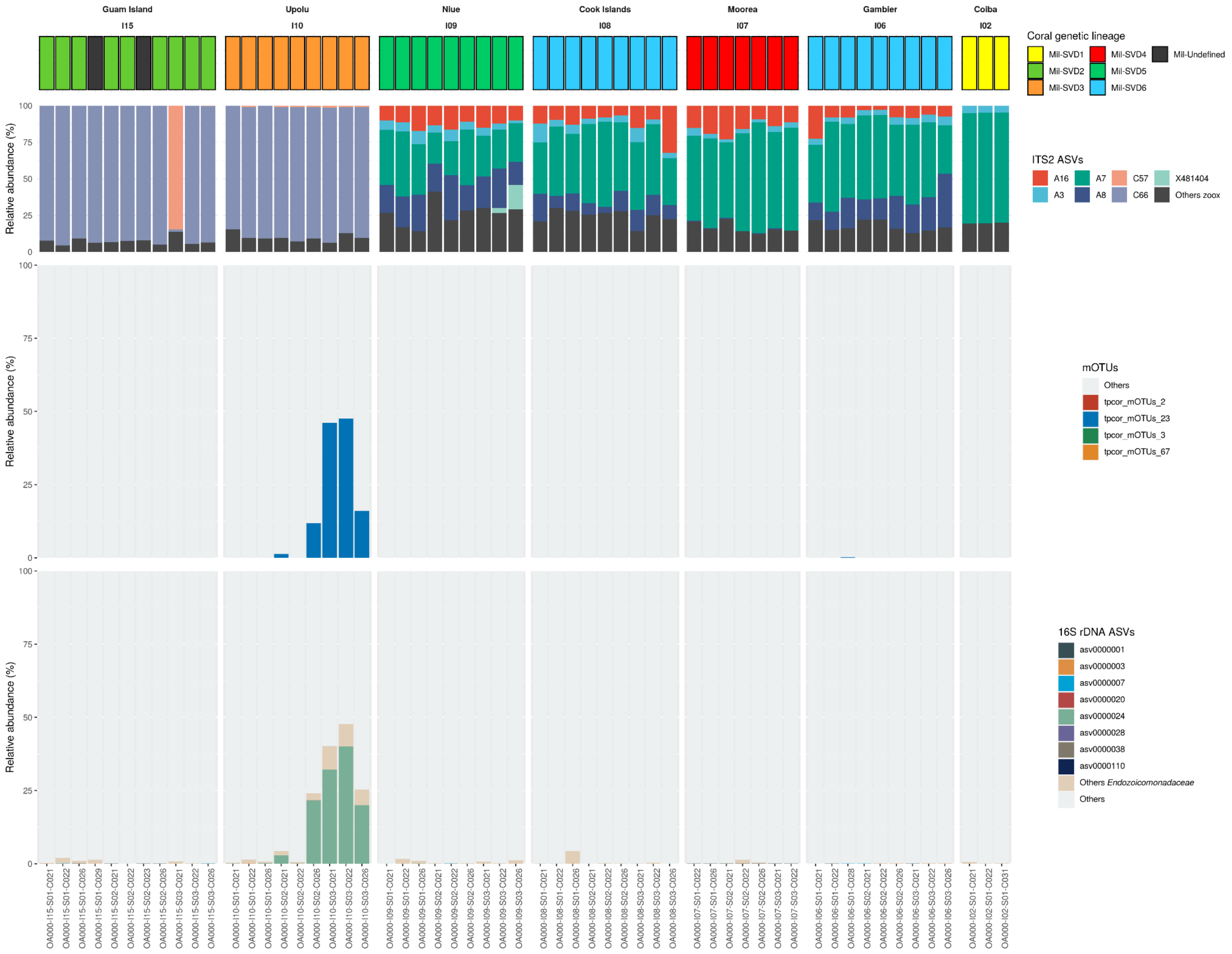

c.

Supplementary Fig. 6. Coral host genetic lineages, and abundances of 16S rRNA ASVs, mOTUs, *Symbiodiniaceae* ITS2 ASVs in *Pocillopora* (a), *Porites* (b) and *Millepora* (c).

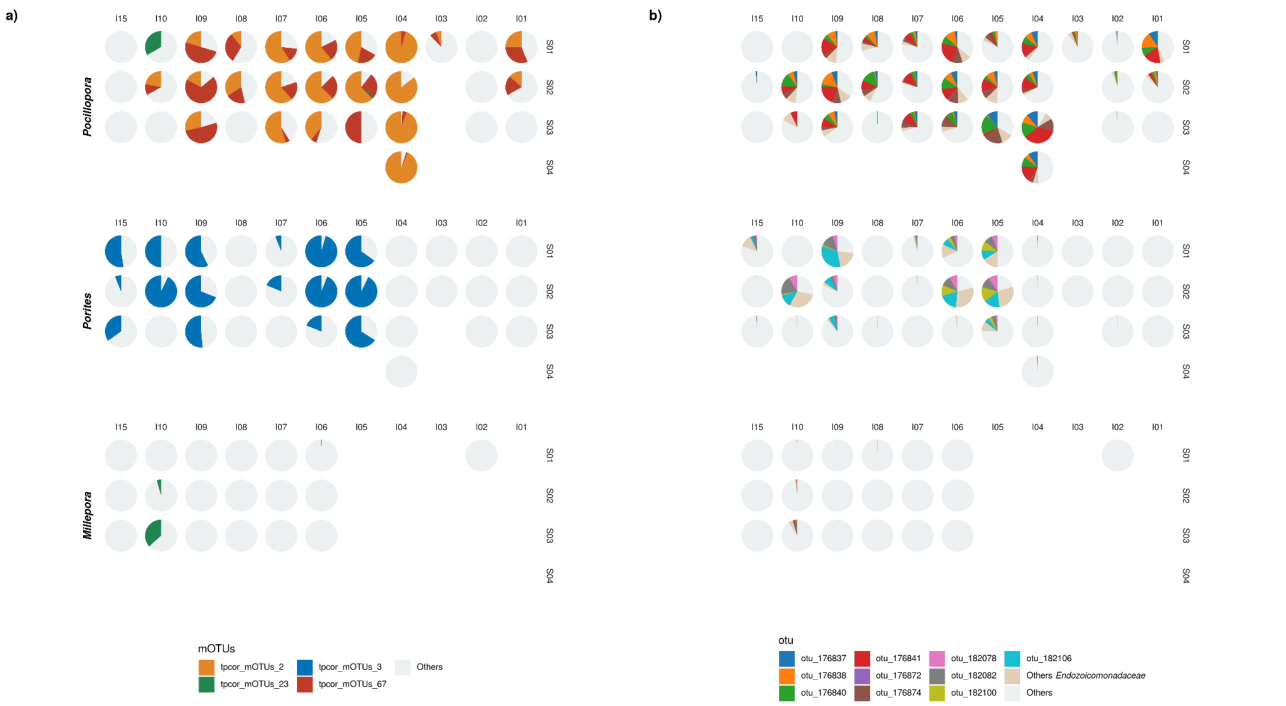

Supplementary Fig. 7. Distribution and abundance profiles of *Endozoicomonadaceae* in 11 islands through the Pacific Ocean in *Pocillopora, Porites* and *Millepora* based on mOTUs (OTUs based on 10 marker genes)(a) and mTAGs (16S rRNA genes extracted from metagenomes)(b).

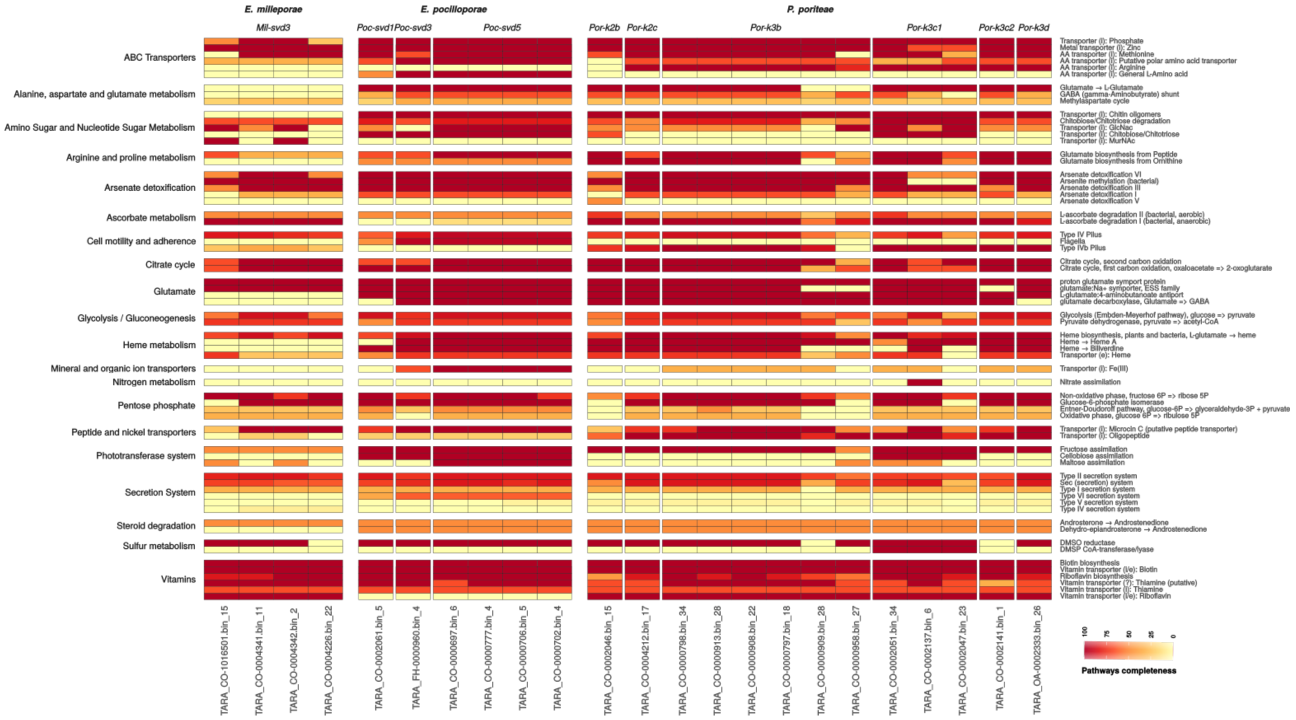

E. milleporae
E. pocilloporae
P. poriteae
Mil-svd3
Poc-svd1
Poc-svd3
Poc-svd5
Por-k2b
Por-k2c
Por-k3b
Por-k3c1
Por-k3c2
Por-k3d
ABC Transporters
Alanine, aspartate and glutamate metabolism
Amino Sugar and Nucleotide Sugar Metabolism
Arginine and proline metabolism
Arsenate detoxification
Ascorbate metabolism
Cell motility and adherence
Citrate cycle
Glutamate
Glycolysis / Gluconeogenesis
Heme metabolism
Mineral and organic ion transporters
Nitrogen metabolism
Pentose phosphate
Peptide and nickel transporters
Phototransferase system
Secretion System
Steroid degradation
Sulfur metabolism
Vitamins
Transporter (i): Phosphate
Metal transporter (i): Zinc
AA transporter (i): Methionine
AA transporter (i): Putative polar amino acid transporter
AA transporter (i): Arginine
AA transporter (i): General L-Amino acid
Glutamate → L-Glutamate
GABA (gamma-Aminobutyrate) shunt
Methylaspartate cycle
Transporter (i): Chitin oligomers
Chitobiose/Chitotriose degradation
Transporter (i): GlcNac
Transporter (i): Chitobiose/Chitotriose
Transporter (i): MurNAc
Glutamate biosynthesis from Peptide
Glutamate biosynthesis from Ornithine
Arsenate detoxification VI
Arsenite methylation (bacterial)
Arsenate detoxification III
Arsenate detoxification I
Arsenate detoxification V
L-ascorbate degradation II (bacterial, aerobic)
L-ascorbate degradation I (bacterial, anaerobic)
Type IV Pilus
Flagella
Type IVb Pilus
Citrate cycle, second carbon oxidation
Citrate cycle, first carbon oxidation, oxaloacetate => 2-oxoglutarate
proton glutamate symport protein
glutamate:Na+ symporter, ESS family
L-glutamate:4-aminobutanoate antiport
glutamate decarboxylase, Glutamate => GABA
Glycolysis (Embden-Meyerhof pathway), glucose => pyruvate
Pyruvate dehydrogenase, pyruvate => acetyl-CoA
Heme biosynthesis, plants and bacteria, L-glutamate → heme
Heme → Heme A
Heme → Biliverdine
Transporter (e): Heme
Transporter (i): Fe(III)
Nitrate assimilation
Non-oxidative phase, fructose 6P => ribose 5P
Glucose-6-phosphate isomerase
Entner-Doudoroff pathway, glucose-6P => glyceraldehyde-3P + pyruvate
Oxidative phase, glucose 6P => ribulose 5P
Transporter (i): Microcin C (putative peptide transporter)
Transporter (i): Oligopeptide
Fructose assimilation
Cellobiose assimilation
Maltose assimilation
Type II secretion system
Sec (secretion) system
Type I secretion system
Type VI secretion system
Type V secretion system
Type IV secretion system
Androsterone → Androstenedione
Dehydro-epiandrosterone → Androstenedione
DMSO reductase
DMSP CoA-transferase/lyase
Biotin biosynthesis
Vitamin transporter (i/e): Biotin
Riboflavin biosynthesis
Vitamin transporter (?): Thiamine (putative)
Vitamin transporter (i): Thiamine
Vitamin transporter (i/e): Riboflavin
TARA_CO-1016501.bin_15
TARA_CO-0004341.bin_11
TARA_CO-0004342.bin_2
TARA_CO-0004226.bin_22
TARA_CO-0002061.bin_5
TARA_FH-0000960.bin_4
TARA_CO-0000697.bin_6
TARA_CO-0000777.bin_4
TARA_CO-0000706.bin_5
TARA_CO-0000702.bin_4
TARA_CO-0002046.bin_15
TARA_CO-0004212.bin_17
TARA_CO-0000798.bin_34
TARA_CO-0000913.bin_28
TARA_CO-0000908.bin_22
TARA_CO-0000797.bin_18
TARA_CO-0000909.bin_28
TARA_CO-0000958.bin_27
TARA_CO-0002051.bin_34
TARA_CO-0002137.bin_6
TARA_CO-0002047.bin_23
TARA_CO-0002141.bin_1
TARA_OA-0002333.bin_26
100
75
50
25
0
Pathways completeness

a.

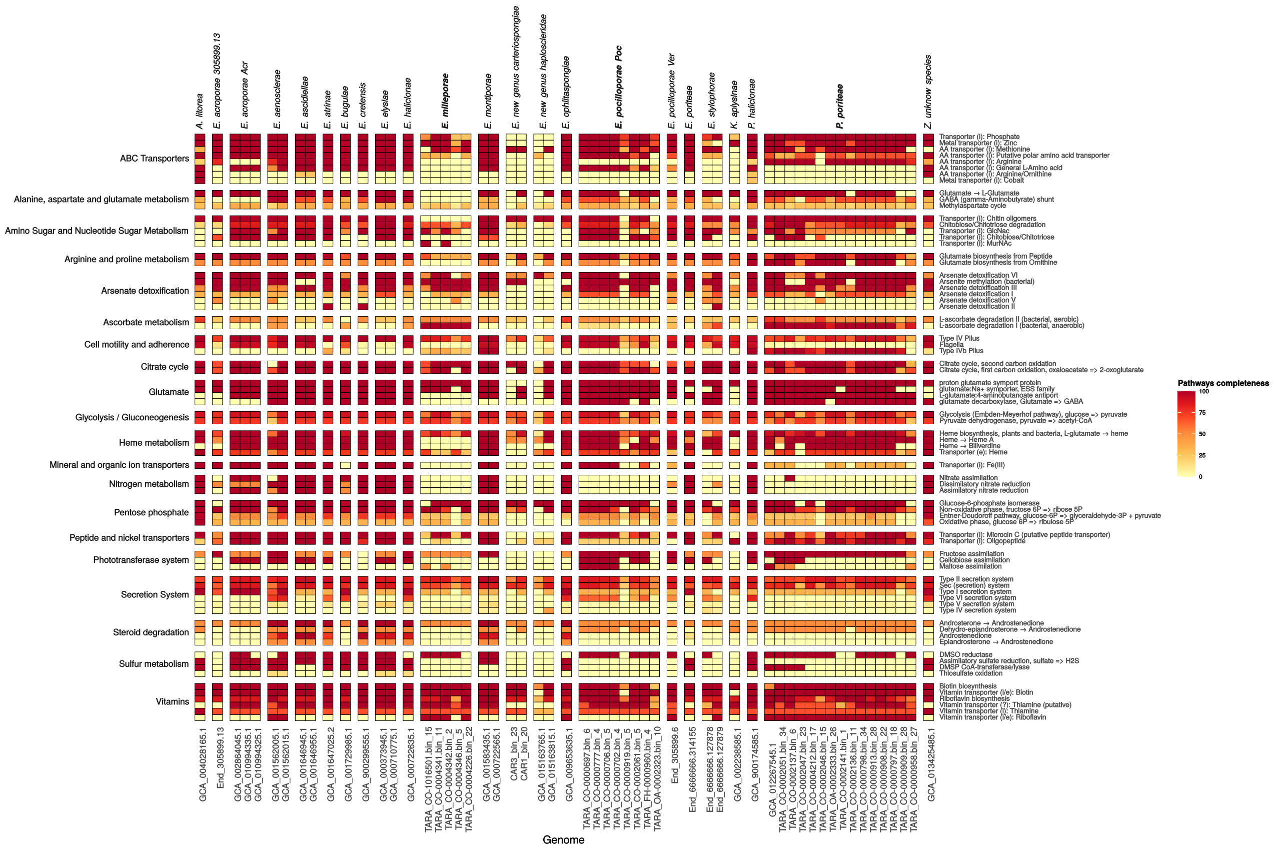

b.

Supplementary Fig. 8. Heatmap showing the potential metabolisms present in the different *Endozoicomonadaceae* species and lineages from this study (a), and compared with MAGs from the literature (b).

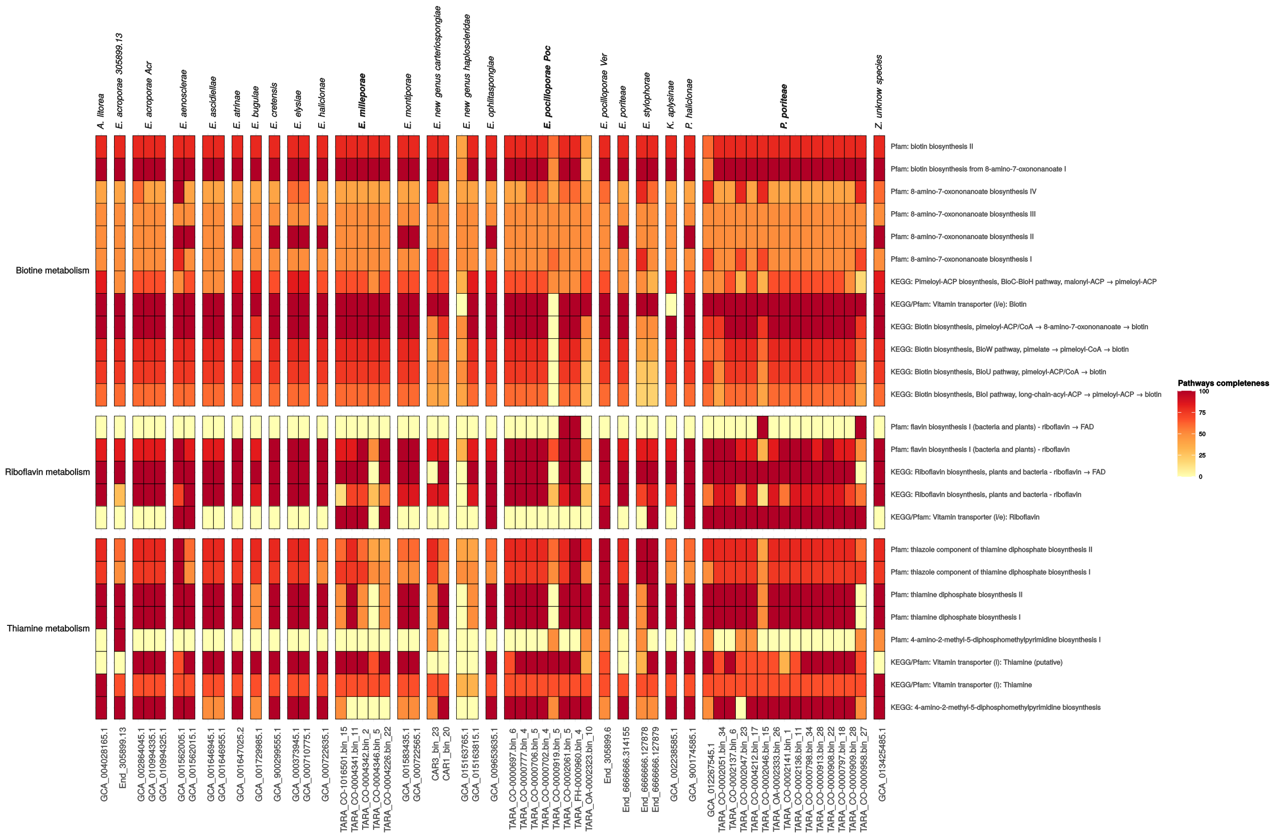
A. litorea
E. acroporae 305899.13
E. acroporae Acr
E. aenoscleraе
E. ascidiellae
E. atrinae
E. bugulae
E. cretensis
E. elysiae
E. haliclonae
E. milleporae
E. montiporae
E. new genus carteriospongiae
E. new genus haploscleridae
E. ophlitaspongiae
E. pocilloporae Poc
E. pocilloporae Ver
E. poriteae
E. stylophorae
K. aplysinae
P. haliclonae
P. poriteae
Z. unknow species
Biotine metabolism
Riboflavin metabolism
Thiamine metabolism
Pfam: biotin biosynthesis II
Pfam: biotin biosynthesis from 8-amino-7-oxononanoate I
Pfam: 8-amino-7-oxononanoate biosynthesis IV
Pfam: 8-amino-7-oxononanoate biosynthesis III
Pfam: 8-amino-7-oxononanoate biosynthesis II
Pfam: 8-amino-7-oxononanoate biosynthesis I
KEGG: Pimeloyl-ACP biosynthesis, BioC-BioH pathway, malonyl-ACP → pimeloyl-ACP
KEGG/Pfam: Vitamin transporter (i/e): Biotin
KEGG: Biotin biosynthesis, pimeloyl-ACP/CoA → 8-amino-7-oxononanoate → biotin
KEGG: Biotin biosynthesis, BioW pathway, pimelate → pimeloyl-CoA → biotin
KEGG: Biotin biosynthesis, BioU pathway, pimeloyl-ACP/CoA → biotin
KEGG: Biotin biosynthesis, BioI pathway, long-chain-acyl-ACP → pimeloyl-ACP → biotin
Pfam: flavin biosynthesis I (bacteria and plants) - riboflavin → FAD
Pfam: flavin biosynthesis I (bacteria and plants) - riboflavin
KEGG: Riboflavin biosynthesis, plants and bacteria - riboflavin → FAD
KEGG: Riboflavin biosynthesis, plants and bacteria - riboflavin
KEGG/Pfam: Vitamin transporter (i/e): Riboflavin
Pfam: thiazole component of thiamine diphosphate biosynthesis II
Pfam: thiazole component of thiamine diphosphate biosynthesis I
Pfam: thiamine diphosphate biosynthesis II
Pfam: thiamine diphosphate biosynthesis I
Pfam: 4-amino-2-methyl-5-diphosphomethylpyrimidine biosynthesis I
KEGG/Pfam: Vitamin transporter (i): Thiamine (putative)
KEGG/Pfam: Vitamin transporter (i): Thiamine
KEGG: 4-amino-2-methyl-5-diphosphomethylpyrimidine biosynthesis
Pathways completeness
100
75
50
25
0
GCA_004028165.1
End_305899.13
GCA_002864045.1
GCA_010994335.1
GCA_010994325.1
GCA_001562005.1
GCA_001562015.1
GCA_001646945.1
GCA_001646955.1
GCA_001647025.2
GCA_001729985.1
GCA_900299555.1
GCA_000373945.1
GCA_000710775.1
GCA_000722635.1
TARA_CO-1016501.bin_15
TARA_CO-0004341.bin_11
TARA_CO-0004342.bin_2
TARA_CO-0004346.bin_5
TARA_CO-0004226.bin_22
GCA_001583435.1
GCA_000722565.1
CAR3_bin_23
CAR1_bin_20
GCA_015163765.1
GCA_015163815.1
GCA_009653635.1
TARA_CO-0000697.bin_6
TARA_CO-0000777.bin_4
TARA_CO-0000706.bin_5
TARA_CO-0000702.bin_4
TARA_CO-0000919.bin_5
TARA_CO-0002061.bin_5
TARA_FH-0000960.bin_4
TARA_OA-0002323.bin_10
End_305899.6
End_6666666.314155
End_6666666.127878
End_6666666.127879
GCA_002238585.1
GCA_900174585.1
GCA_012267545.1
TARA_CO-0002051.bin_34
TARA_CO-0002137.bin_6
TARA_CO-0002047.bin_23
TARA_CO-0004212.bin_17
TARA_CO-0002046.bin_15
TARA_OA-0002333.bin_26
TARA_CO-0002141.bin_1
TARA_CO-0002136.bin_11
TARA_CO-0000798.bin_34
TARA_CO-0000913.bin_28
TARA_CO-0000908.bin_22
TARA_CO-0000797.bin_18
TARA_CO-0000909.bin_28
TARA_CO-0000958.bin_27
GCA_013425485.1

a.

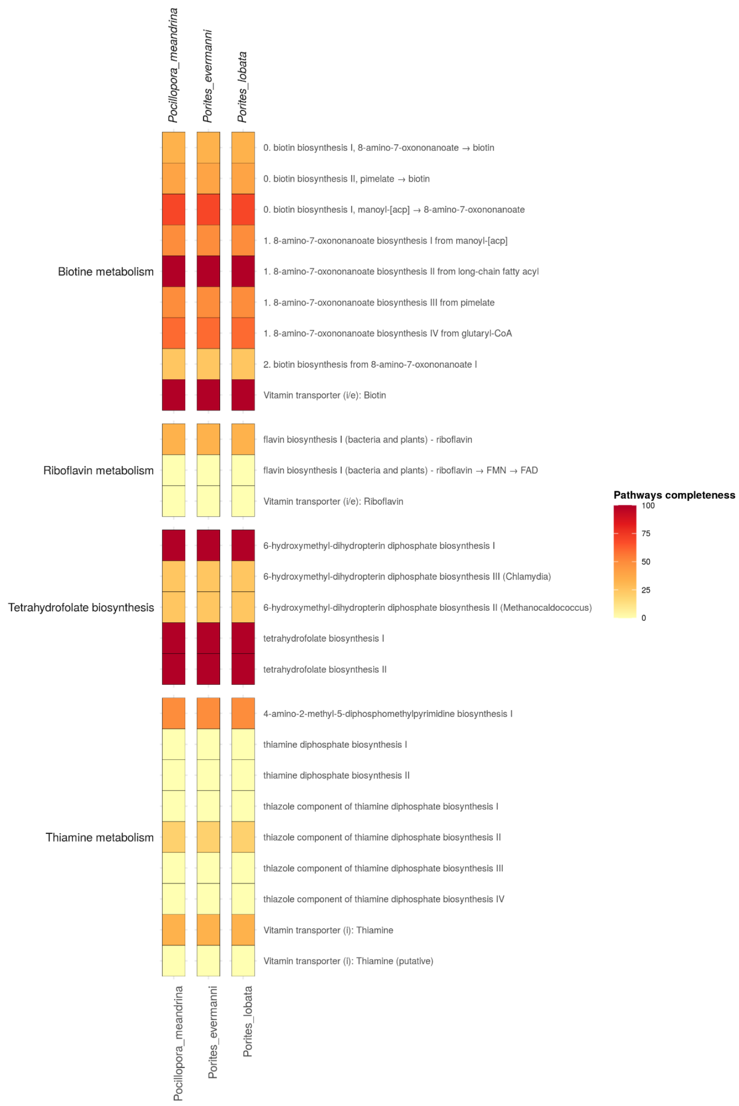

b.

Supplementary Fig. 9. Heatmap showing the presence of genes associated to the metabolisms of biotin (vitamin B7), riboflavin (B2), folate (B9), and thiamine (B1) in *Endozoicomonadaceae* MAGs from this study (TARA_) and others (a), and in the coral hosts (b). Colours indicate the pathway completeness.

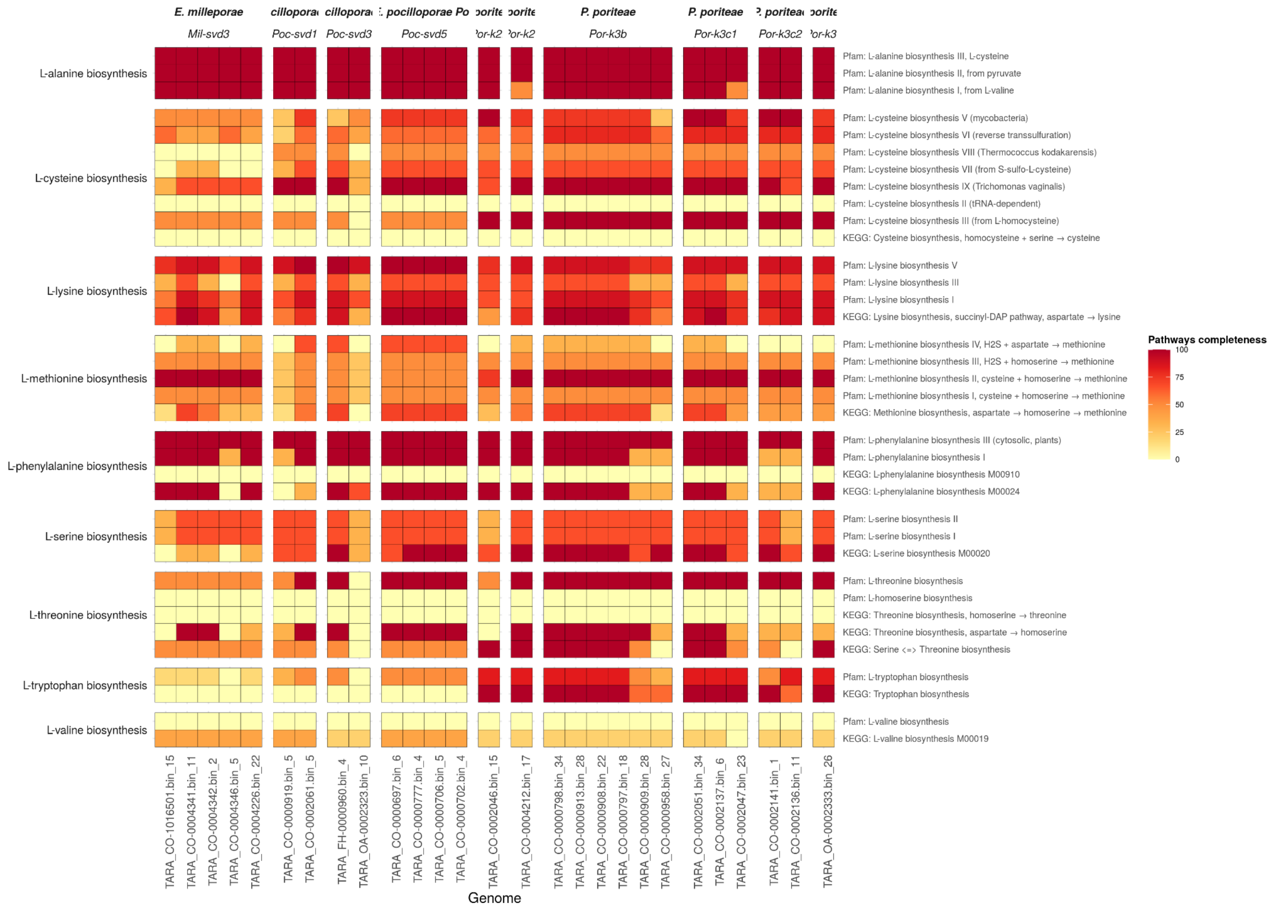
E. milleporae
Mil-svd3
Poc-svd1
Poc-svd3
Poc-svd5
P. poriteae
Por-k3b
P. poriteae
Por-k3c1
Por-k3c2
L-alanine biosynthesis
L-cysteine biosynthesis
L-lysine biosynthesis
L-methionine biosynthesis
L-phenylalanine biosynthesis
L-serine biosynthesis
L-threonine biosynthesis
L-tryptophan biosynthesis
L-valine biosynthesis
Pfam: L-alanine biosynthesis III, L-cysteine
Pfam: L-alanine biosynthesis II, from pyruvate
Pfam: L-alanine biosynthesis I, from L-valine
Pfam: L-cysteine biosynthesis V (mycobacteria)
Pfam: L-cysteine biosynthesis VI (reverse transsulfuration)
Pfam: L-cysteine biosynthesis VIII (Thermococcus kodakarensis)
Pfam: L-cysteine biosynthesis VII (from S-sulfo-L-cysteine)
Pfam: L-cysteine biosynthesis IX (Trichomonas vaginalis)
Pfam: L-cysteine biosynthesis II (tRNA-dependent)
Pfam: L-cysteine biosynthesis III (from L-homocysteine)
KEGG: Cysteine biosynthesis, homocysteine + serine → cysteine
Pfam: L-lysine biosynthesis V
Pfam: L-lysine biosynthesis III
Pfam: L-lysine biosynthesis I
KEGG: Lysine biosynthesis, succinyl-DAP pathway, aspartate → lysine
Pfam: L-methionine biosynthesis IV, H2S + aspartate → methionine
Pfam: L-methionine biosynthesis III, H2S + homoserine → methionine
Pfam: L-methionine biosynthesis II, cysteine + homoserine → methionine
Pfam: L-methionine biosynthesis I, cysteine + homoserine → methionine
KEGG: Methionine biosynthesis, aspartate → homoserine → methionine
Pfam: L-phenylalanine biosynthesis III (cytosolic, plants)
Pfam: L-phenylalanine biosynthesis I
KEGG: L-phenylalanine biosynthesis M00910
KEGG: L-phenylalanine biosynthesis M00024
Pfam: L-serine biosynthesis II
Pfam: L-serine biosynthesis I
KEGG: L-serine biosynthesis M00020
Pfam: L-threonine biosynthesis
Pfam: L-homoserine biosynthesis
KEGG: Threonine biosynthesis, homoserine → threonine
KEGG: Threonine biosynthesis, aspartate → homoserine
KEGG: Serine <=> Threonine biosynthesis
Pfam: L-tryptophan biosynthesis
KEGG: Tryptophan biosynthesis
Pfam: L-valine biosynthesis
KEGG: L-valine biosynthesis M00019
Pathways completeness
100
75
50
25
0
TARA_CO-1016501.bin_15
TARA_CO-0004341.bin_11
TARA_CO-0004342.bin_2
TARA_CO-0004346.bin_5
TARA_CO-0004226.bin_22
TARA_CO-0000919.bin_5
TARA_CO-0002061.bin_5
TARA_FH-0000960.bin_4
TARA_OA-0002323.bin_10
TARA_CO-0000697.bin_6
TARA_CO-0000777.bin_4
TARA_CO-0000706.bin_5
TARA_CO-0000702.bin_4
TARA_CO-0002046.bin_15
TARA_CO-0004212.bin_17
TARA_CO-0000798.bin_34
TARA_CO-0000913.bin_28
TARA_CO-0000908.bin_22
TARA_CO-0000797.bin_18
TARA_CO-0000909.bin_28
TARA_CO-0000958.bin_27
TARA_CO-0002051.bin_34
TARA_CO-0002137.bin_6
TARA_CO-0002047.bin_23
TARA_CO-0002141.bin_1
TARA_CO-0002136.bin_11
TARA_OA-0002333.bin_26
Genome

a.

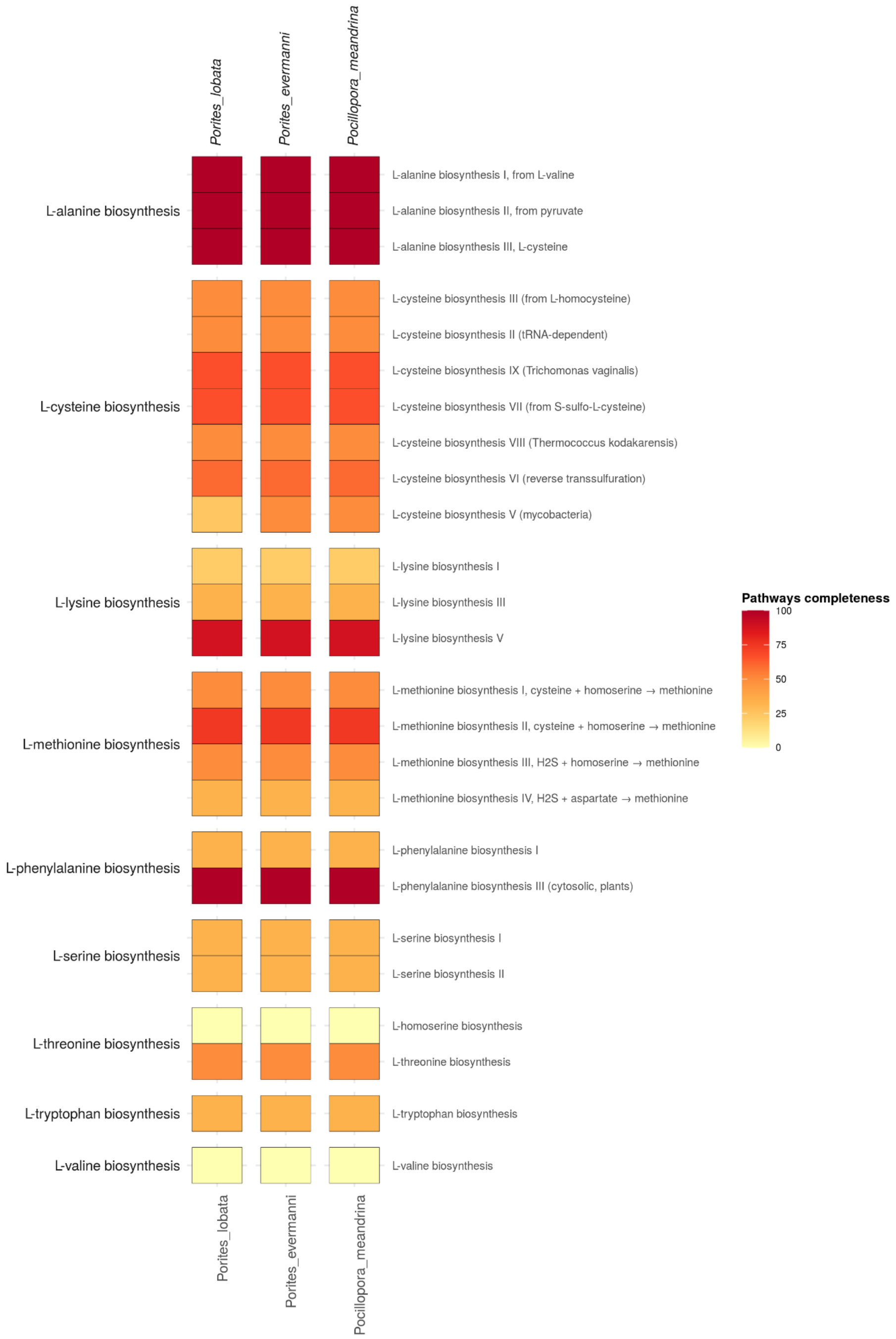

b.

Supplementary Fig. 10. Heatmap showing the presence of genes associated to production of different amino acids in *Endozoicomonadaceae* MAGs from this study (a) and from their coral hosts (b). Colours indicate the pathway completeness.

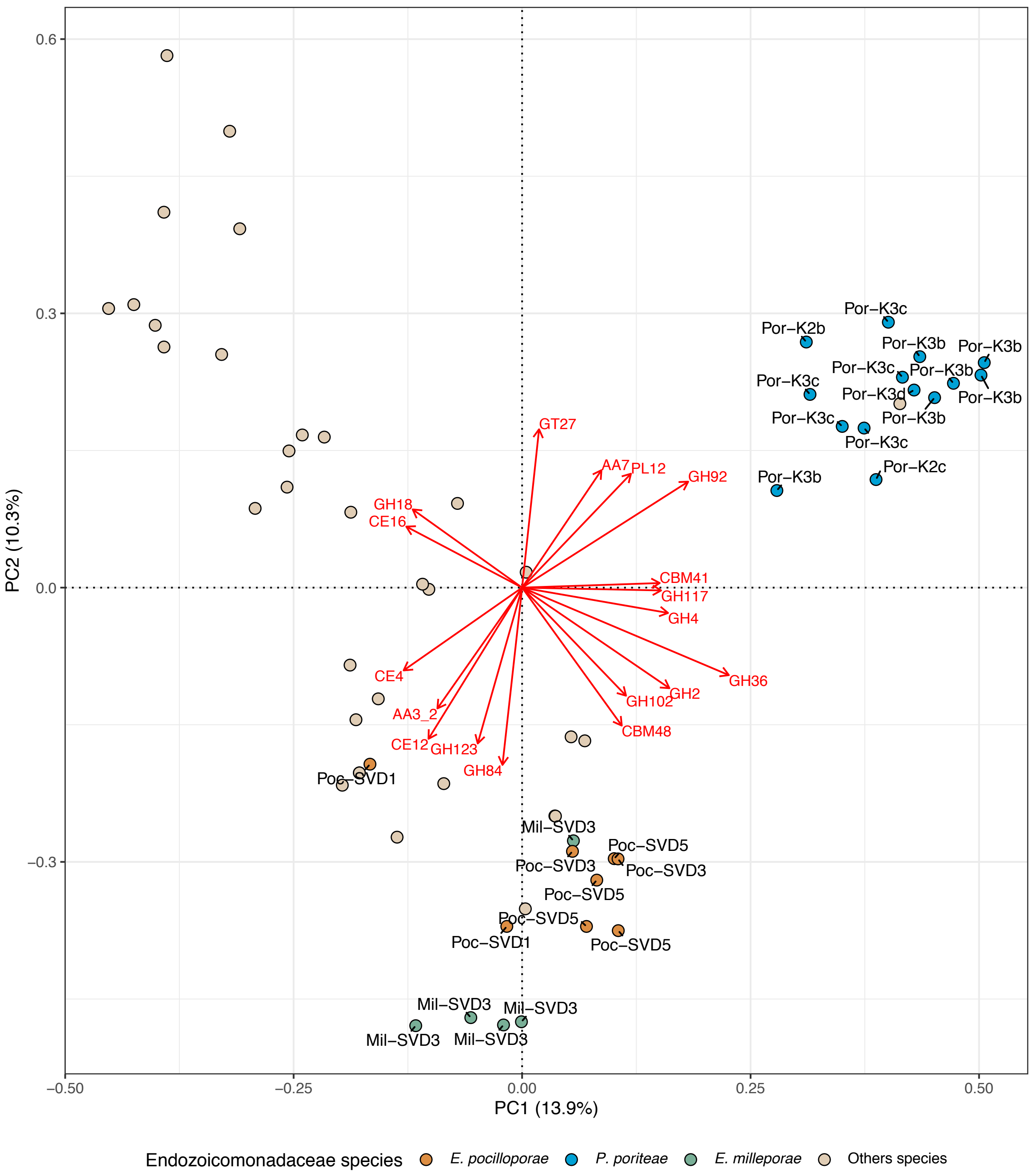

Supplementary Fig. 11: Transformation-based principal component analysis (PCA) based on carbohydrate-active enzymes (CAZymes) composition of the *Endozoicomonadaceae* MAGs. Only CAZymes family with vectors longer than the equilibrium contribution are displayed.

Supplementary discussion

Endozoicomonadaceae phylogeny

The 24 *Endozoicomonadaceae* MAGs constructed from our cross-Pacific sampling of coral reefs allowed us to describe three new *Endozoicomonadaceae* species: *Ca. Endozoicomonas milleporae*, *Ca. Endozoicomonas pocilloporae,* and *Ca. Parendozoicomonas poritae*. Following established rules, the name of the species corresponds to the host coral genus. The three new *Endozoicomonadaceae* species add to the 16 species of *Endozoicomonadaceae* defined to date[1]. Within the *Endozoicomonadaceae* family, four species that we describe fall within two genera: *Parendozoicomonas and Endozoicomonas*. The *Endozoicomonas* genus was first described by Kurahashi and Yokota[2] as a γ-proteobacterium isolated from the sea slug *Elysia ornata,* named *Endozoicomas elysicola.* This new genera appeared to be related to the genus *Zooshikella*. In the following years, 11 new species of *Endozoicomonas* were described after being isolated from different invertebrate marine hosts such as ascidians[3], bivalves[4], coral[5–8], fish[9] and sponges[10–13]. The genus *Parendozoicomonas* was recently proposed by Bartz *et al.*[14] to include a new species (*Parendozoicomonas haliclonae)* including two new strains (S-B4-1UT and JOB-63a) isolated from a marine sponge of the genus *Haliclona*, based on 16S rRNA gene and whole genome analysis. Our study adds a second species to the *Parendozoicomonas* and reclassifies the MAG Plut_88861 from a *Porites lutea*[15] as *Parendozoicomonas* (originally classified as *Endozoicomonas*). The *Endozoicomonadaceae* family also includes the *Kistimonas* genus that was not detected among our MAGs. *Kistimonas* was first described by Choi *et al.*[16] with *Kistimonas asteriae,* isolated from the starfish *Asterias amurensis.* A total of four new species of *Kistimonas* were subsequently described from clams[17], ribbon worm[18], marine sponge[19] and sandworm[20]. Lately, a fourth genera was proposed within the *Endozoicomonadaceae* family*: Sansalvadorimonas* first isolated from the marine sponge *Verongula gigantea*[11]. Recently, Liao *et al.*[21] proposed to transfer the family *Endozoicomonadaceae* to a novel family, which would be named *Zooshikellaceae,* comprising four genera: *Endozoicomonas, Kistimonas, Parendozoicomonas* and *Zooshikella.* The reclassification was supported by core-genes, genes markers and 16 rRNA analysis. The *Zooshikella* genus does not comprise host associated bacteria as it comprises at the moment three species *Z. ganghwensis*[22], *Z. rubidus*[23] *and Z. marina*[24], which were isolated from sediments, beach sand and tidal flat sediment. The new classification remains a matter of debate since the Genome Taxonomy Database (GTDB) includes the genera *Aestuariirhabdus* and *Zooshikella* in the novel family *Zooshikellaceae* and Li *et al.* did not include, *Spartinivicinus* and *Aestuariirhabdus* in their phylogenetic study[1]. Our study support the existence of a well-defined bacteria family named *Endozoicomonadaceae,* which is now composed of four genera, *Endozoicomonas* (14 species), *Parendozoicomonas* (two species), *Sansalvadorimonas* (one species) and *Kistimonas* (three species).

Supplementary References

1. Huang, Z., Su, P. & Lai, Q. Proposal of zooshikellaceae fam. Nov. to accommodate the genera zooshikella and spartinivicinus and reclassification of zooshikella marina as a later heterotypic synonym of zooshikella ganghwensis based on whole genome sequence analysis. *International Journal of Systematic and Evolutionary Microbiology* **71**, 005055–005055 (2021).
2. Kurahashi, M. & Yokota, A. Endozoicomonas elysicola gen. nov., sp. nov., a γ-proteobacterium isolated from the sea slug Elysia ornata. *Systematic and Applied Microbiology* **30**, 202–206 (2007).
3. Schreiber, L. *et al.* Endozoicomonas are specific, facultative symbionts of sea squirts. *Frontiers in Microbiology* **7**, (2016).
4. Hyun, D. W. *et al.* Endozoicomonas atrinae sp. nov., isolated from the intestine of a comb pen shell Atrina pectinata. *International Journal of Systematic and Evolutionary Microbiology* **64**, 2312–2318 (2014).
5. Bayer, T. *et al.* The microbiome of the red sea coral stylophora pistillata is dominated by tissue-associated endozoicomonas bacteria. *Applied and Environmental Microbiology* **79**, 4759–4762 (2013).
6. Pike, R. E., Haltli, B. & Kerr, R. G. Description of Endozoicomonas euniceicola sp. nov. and Endozoicomonas gorgoniicola sp. nov., bacteria isolated from the octocorals Eunicea fusca and Plexaura sp., and an emended description of the genus Endozoicomonas. *International Journal of Systematic and Evolutionary Microbiology* **63**, 4294–4302 (2013).
7. Sheu, S. Y. *et al.* Endozoicomonas acroporae sp. nov., isolated from Acropora coral. *International Journal of Systematic and Evolutionary Microbiology* **67**, 3791–3797 (2017).
8. Yang, S. H. *et al.* Long-term survey is necessary to reveal various shifts of microbial composition in corals. *Frontiers in Microbiology* **8**, (2017).
9. Katharios, P. *et al.* Environmental marine pathogen isolation using mesocosm culture of sharpsnout seabream: Striking genomic and morphological features of novel Endozoicomonas sp. *Scientific Reports* **5**, (2015).
10. Appolinario, L. R. *et al.* Description of Endozoicomonas arenosclerae sp. nov. using a genomic taxonomy approach. *Antonie van Leeuwenhoek, International Journal of General and Molecular Microbiology* **109**, 431–438 (2016).
11. Goldberg, S. R., Haltli, B. A., Correa, H. & Kerr, R. G. Description of sansalvadorimonas verongulae gen. Nov., sp. nov., a gammaproteobacterium isolated from the marine sponge Verongula gigantea. *International Journal of Systematic and Evolutionary Microbiology* **68**, 2006–2014 (2018).
12. Alex, A. & Antunes, A. Comparative Genomics Reveals Metabolic Specificity of Endozoicomonas Isolated from a Marine Sponge and the Genomic Repertoire for Host-Bacteria Symbioses. *Microorganisms* **7**, (2019).
13. Nishijima, M., Adachi, K., Katsuta, A., Shizuri, Y. & Yamasato, K. Endozoicomonas numazuensis sp. nov., a gammaproteobacterium isolated from marine sponges, and emended description of the genus Endozoicomonas Kurahashi and Yokota 2007. *International Journal of Systematic and Evolutionary Microbiology* **63**, 709–714 (2013).
14. Bartz, J. O. *et al.* Parendozoicomonas haliclonae gen. nov. sp. nov. isolated from a marine sponge of the genus Haliclona and description of the family Endozoicomonadaceae fam. nov. comprising the genera Endozoicomonas, Parendozoicomonas, and Kistimonas. *Systematic and Applied Microbiology* **41**, 73–84 (2018).
15. Robbins, S. J. *et al.* A genomic view of the reef-building coral Porites lutea and its microbial symbionts. *Nature Microbiology* **4**, 2090–2100 (2019).
16. Choi, E. J., Kwon, H. C., Sohn, Y. C. & Yang, H. O. Kistimonas asteriae gen. nov., sp. nov.,

a gammaproteobacterium isolated from Asterias amurensis. *International Journal of Systematic and Evolutionary Microbiology* **60**, 938–943 (2010).
17. Lee, J. *et al.* Kistimonas scapharcae sp. nov., isolated from a dead ark clam (Scapharca broughtonii), and emended description of the genus Kistimonas. *International Journal of Systematic and Evolutionary Microbiology* **62**, 2865–2869 (2012).
18. Beleneva, I. A., Magarlamov, T. Y. & Kukhlevsky, A. D. Characterization, identification, and screening for tetrodotoxin production by bacteria associated with the ribbon worm (Nemertea) Cephalotrix simula (Ivata, 1952). *Microbiology 2014 83:3* **83**, 220–226 (2014).
19. Slaby, B. M., Hackl, T., Horn, H., Bayer, K. & Hentschel, U. Metagenomic binning of a marine sponge microbiome reveals unity in defense but metabolic specialization. *The ISME Journal 2017 11:11* **11**, 2465–2478 (2017).
20. Christopher Ellis, J. *et al.* Kistimonas alittae sp. nov., a gammaproteobacterium isolated from the marine annelid Alitta succinea. *International Journal of Systematic and Evolutionary Microbiology* **69**, 235–240 (2019).
21. Liao, H., Lin, X., Li, Y., Qu, M. & Tian, Y. Reclassification of the Taxonomic Framework of Orders Cellvibrionales, Oceanospirillales, Pseudomonadales , and Alteromonadales in Class Gammaproteobacteria through Phylogenomic Tree Analysis. *mSystems* **5**, (2020).
22. Yi, H., Chang, Y. H., Oh, H. W., Bae, K. S. & Chun, J. Zooshikella ganghwensis gen. nov., sp. nov., isolated from tidal flat sediments. *International Journal of Systematic and Evolutionary Microbiology* **53**, 1013–1018 (2003).
23. Lee, J. S. *et al.* Exceptional production of both prodigiosin and cycloprodigiosin as major metabolic constituents by a novel marine bacterium, Zooshikella rubidus S1-1. *Applied and Environmental Microbiology* **77**, 4967–4973 (2011).
24. Ramaprasad, E. V. V., Bharti, D., Sasikala, C. & Ramana, C. V. Zooshikella marina sp. nov. a cycloprodigiosinand prodigiosin-producing marine bacterium isolated from beach sand. *International Journal of Systematic and Evolutionary Microbiology* **65**, 4669–4673 (2015).
