# Supplementary material for: Ecology of Endozoicomonadaceae in three coral genera across the Pacific Ocean
Source: Nat Commun. 2023 Jun 1;14:3037. doi: 10.1038/s41467-023-38502-9 (PMC10235432; doi:10.1038/s41467-023-38502-9)
Supplement: Supplementary file 3 — Description of Additional Supplementary Files [file 41467_2023_38502_MOESM3_ESM.pdf]

Description of Additional Supplementary Files

File Name: Supplementary Data 1
Description: Characteristics of the Endozoicomonadaceae genomes reconstructed in this study and from the literature

File Name: Supplementary Data 2
Description: List of CAZy and their family characteristics. CAZy were identified by PCA analyse of CAZy abundance in Endozoicomonadaceae MAGs (Supplementary Fig. 11).
